# Cerebral Activity Associated with Transient Sleep-Facilitated Reduction in Motor Memory Vulnerability to Interference

**DOI:** 10.1038/srep34948

**Published:** 2016-10-11

**Authors:** Geneviève Albouy, Bradley R. King, Christina Schmidt, Martin Desseilles, Thien Thanh Dang-Vu, Evelyne Balteau, Christophe Phillips, Christian Degueldre, Pierre Orban, Habib Benali, Philippe Peigneux, André Luxen, Avi Karni, Julien Doyon, Pierre Maquet, Maria Korman

**Affiliations:** 1 Cyclotron Research Centre, University of Liège, Liège, Belgium; 2 Functional Neuroimaging Unit, Centre de Recherche de l’Institut Universitaire de Gériatrie de Montreal, University of Montreal, Montreal, Canada; 3 Movement Control and Neuroplasticity Research Group, Department of Kinesiology, KU Leuven, Leuven, Belgium; 4 PERFORM Center and Center for Studies in Behavioral Neurobiology, Department of Exercise Science, Concordia University, Montreal, Canada; 5 Unité Mixte de Recherche-S 678, Institut National de la Santé et de la Recherche Médicale/University of Paris 6, Centre Hospitalier Universitaire Pitié-Salpêtrière, Paris, France; 6 UR2NF - Neuropsychology and Functional Neuroimaging Research Unit affiliated at CRCN - Center for Research in Cognition and Neurosciences, Neurosciences Institute, Université Libre de Bruxelles, Brussels, Belgium; 7 Laboratory for Functional Brain Imaging and Learning Research, The Brain-Behavior Center, University of Haifa, Haifa, Israel; 8 Occupational Therapy Department, Faculty of Social Welfare & Health Sciences, University of Haifa, Haifa, Israel

## Abstract

Motor memory consolidation is characterized, in part, by a sleep-facilitated decrease in susceptibility to subsequent interfering experiences. Surprisingly, the cerebral substrates supporting this phenomenon have never been examined. We used fMRI to investigate the neural correlates of the influence of sleep on interference to motor memory consolidation. Healthy young adults were trained on a sequential motor task, and subsequently practiced a second competing sequence after an interval including diurnal sleep or wakefulness. Participants were then retested on the initial sequence 8 h and 24 h (including nocturnal sleep) after training. Results demonstrated that a post-training nap significantly protected memory against interference at 8 h and modulated the link between cerebral activity and behavior, such that a smaller post-interference decrease in cortico-striatal activity was associated with better performance. Interestingly, the protective effect of a nap was only transitory, as both groups performed similarly at 24 h. Activity in cortico-striatal areas that was disrupted during the day, presumably due to interference and accentuated in the absence of a nap, was restored overnight. Altogether, our findings offer the first evidence that cortico-striatal areas play a critical role in the transient sleep-facilitated reduction in motor memory vulnerability and in the overnight restoration of previously degraded memories.

Motor memory consolidation is the physiological process by which a newly acquired memory trace is transformed into a more long-lasting and robust form^1^. This process is often characterized by two non-mutually exclusive phenomena: 1) maintenance or enhancement – but not substantial deterioration – of performance over an interval including no further practice^2^; and, 2) resistance to interference from potential competing memories, usually expressed by performance maintenance after an interfering episode^3^. Interestingly, there is now considerable evidence that sleep favors the development of the first behavioral determinant and consequently facilitates motor sequence memory retention as compared to wakefulness. Specifically, post-learning sleep has been shown to promote maintenance^4^,^5^,^6^ or enhancement^6^,^7^,^8^,^9^,^10^,^11^,^12^,^13^,^14^,^15^,^16^,^17^,^18^,^19^,^20^ of motor performance (typically observed for speed rather than accuracy^6^,^16^,^19^,^20^), as compared to wakefulness. However, only one study up to date has investigated the effect of sleep on interference processes; results indicated that post-learning sleep protects newly acquired motor memories against the deleterious effect of interference caused by the practice of a competing motor task^11^.

At the cerebral level, motor sequence memory consolidation in the absence of potential interfering tasks, as well as the influence of sleep on this process, has been extensively investigated^14^,^16^,^17^,^18^,^20^,^21^,^22^. A recent model integrating these different findings proposed that motor sequence learning involves a complex interaction between at least two cerebral systems, i.e., cortico-striatal and cortico-hippocampal networks^2^. According to this model, these different systems would support separate but interacting consolidation processes. The hippocampal system favors sleep-dependent motor memory consolidation processes, whereas the striatal system appears to support more of a time-dependent processing of the memory trace^6^, that may nevertheless be modulated by sleep via interactions with the hippocampal system^2^. Successful retention of motor sequence memories is then thought to be supported by a cooperative interplay between the striato-motor and hippocampo-cortical networks^2^,^17^. Surprisingly, however, the neural correlates of interference to motor memory consolidation and the influence of sleep on these correlates have never been characterized. This knowledge gap is striking as understanding how the brain deals with the plethora of motor tasks – which often share common attributes – necessary for everyday functioning is of the utmost importance.

The aim of this study was thus to investigate the neural correlates of the influence of sleep on interference to motor sequence memory consolidation. To do so, we adapted a previously published protocol^11^ to fMRI and scanned participants during six separate sessions in which they were either *trained* or *tested* on an explicitly known sequence of finger movements. Specifically, participants were trained on a first sequence (Sequence A, Fig. 1) and subsequently practiced a second, competing motor sequence (Sequence B) after an interval including either diurnal sleep (NAP, n = 28) or wakefulness (NONAP, n = 21). Performance on the initially learned sequence was retested 8 h and 24 h (including a full night of sleep) after training in order to measure the influence of diurnal sleep before interfering practice on consolidation of the previously learned motor sequence across same- and next-day intervals. It is important to note that, in the present study, it is only assumed that the practice of a second sequence after initial training will interference with consolidation processes (as shown in the extant literature^11^,^23^,^24^,^25^) as no control groups with no interference were included. At the behavioral level, we predicted that motor performance would be impaired after practice of a second competing sequence if napping was not allowed after training whereas a post-training nap would alleviate this deficit, thus confirming that sleep protects motor memories against deleterious effect of interference^11^. At the cerebral level, based on previous neuroimaging work^2^, we hypothesized that, in the absence of a post-training nap, practicing a competing sequence after learning would interfere with activity in consolidation-relevant neural networks, specifically striatal- and hippocampo-cortical areas and therefore alter performance retested during both same- and next-day retests. In contrast, we expected that, after a protective post-training nap, activity within these networks would not be altered (or to a lesser extent); and, specifically, increases in activity within these cerebral areas – and more particularly in the hippocampo-cortical network due to its tight link to sleep-related processes – would support intact motor sequence memory consolidation processes.

## Results

**Figure 1:**
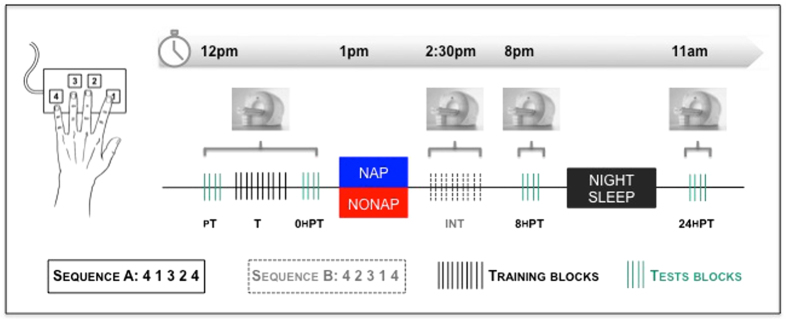
Experimental design. Subjects were trained on Sequence A (T session) and Sequence B (INT session). Performance on Sequence A was tested on several sessions: pre-training (pT), immediately (less than 5 minutes) after training (0 hPT), 8 h (8 hPT) and 24 h (24 hPT) after the end of training. Subjects were divided in two groups according to whether they were afforded a 90-min nap opportunity (NAP) or stayed awake (NONAP) after initial training on sequence A, i.e. before the interfering training session. Note that all subjects completed the interference task and that only the data related to the test blocks (green) are reported in this paper.

Results related to measures of sleep and vigilance prior to and during the experiment are reported in Table 1. Importantly, the two groups did not statistically differ in any of the measures. Subjects in the NAP group experienced on average 60 minutes of sleep (ranging from to 27 to 82 minutes). Due to the stringent NONAP condition, some subjects showed difficulty to remain awake during the 90-minute episode and were therefore excluded if they presented more than one 30s-sleep epoch (see methods). As the goal of the present study is to investigate the influence of sleep on the time course of consolidation processes after interference, only the behavioral and brain-imaging data related to the *test sessions* on the learned sequence are reported in the present paper (i.e., pT, 0 hPT, 8 hPT and 24 hPT indicated in green in Fig. 1).

### Behavioral results

Behavioral analyses were performed on performance speed (mean time to perform a correct sequence per block of practice) and accuracy (number of correct sequences per block of practice) recorded during the 4 test sessions on sequence A (pT, 0 hPT, 8 hPT and 24 hPT).

[Note that training performance on sequence B did not differ between the NAP and NONAP groups in terms of both speed and accuracy (repeated measure ANOVA including 10 blocks of training as within-subject factor and group as between-subject factor showed non-significant main effect of group for both speed (F(1, 47) = 1.37, p = 0.24) and accuracy (F(1, 47) = 0.31, p = 0.57)].

### Speed

**Figure 2:**
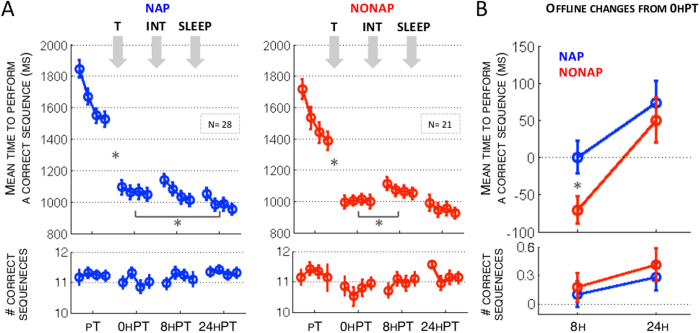
(**A**) Performance speed (upper panel) and accuracy (lower panel) during the four test sessions on Sequence A (pT, 0 hPT, 8 hPT and 24 hPT) preceding and following periods of training on Sequence A (T), napping or wakefulness (NAP vs. NONAP), interfering training on Sequence B (INT) and nocturnal sleep (SLEEP). Bars represent SEM. *Represents significant between-session effects (p < 0.05) within each group. (**B**) Offline changes in performance speed (upper panel) and accuracy (lower panel) at 8 h (average of the difference in performance between 0 hT vs. 8 hPT) and 24 h (average of the difference in performance between 0 hPT vs. 24 hPT). Bars represent SEM. *Represents significant between-group effects (p < 0.05).

We conducted a repeated-measures ANOVA on performance speed during the 4 test sessions (pT, 0 hPT, 8 hPT and 24 hPT, 4 blocks in each session) with block and session as the within-subject factors and group (NAP vs. NONAP) as the between-subject factor. Results revealed significant main effects of session (F(3, 141) = 257.77, p < 0.001) and block (F(3, 141) = 59.46, p < 0.001), indicating that performance improved, unsurprisingly, with practice across blocks and sessions in both groups. No significant group effect was observed (F(1, 47) = 1.31, p = 0.29). The block by group (F(3, 141) = 1.36, p = 0.25) and block by group by session (F(9, 423) = 0.39, p = 0.93) interactions were not significant, indicating that the two groups did not differ in online performance changes across the 4 blocks of practice in the various sessions. Importantly, the session by group interaction was significant (F(3, 141) = 2.59, p = 0.05), suggesting that between-session changes in performance differed between the two groups. We then conducted appropriate within- and between-group analyses to decompose this significant interaction (Fig. 2A,B upper panels, respectively).

Performance significantly improved between pT and 0 hPT in both groups (pT vs. 0 hPT; NAP, F(1, 47) = 235.53, p < 0.001; NONAP, F(1, 47) = 139.73, p < 0.001, Fig. 2A, upper panel), indicating that the training phase significantly improved performance on the task. The two groups did not differ in the improvements from pT to 0 hPT (F(1, 47) = 1.23, p = 0.27).

Eight hours after training, within-group analyses demonstrated performance maintenance in the NAP group (0 hPT vs. 8 hPT; F(1, 47) = 0.001, p = 0.97). Yet there was a significant deterioration in performance in the NONAP group as compared to 0 hPT (F(1, 47) = 10.09, p = 0.002, Fig. 2A, upper panel). Importantly, these effects were significantly different between groups (F(1, 47) = 5.88, p = 0.01, Fig. 2B, upper panel, Changes 8 h), suggesting that napping after training protected performance against deterioration presumably mediated by interference processes 8 h after training.

Significant improvements in performance were observed at 24 h as compared to post-training in the NAP group but not in the NONAP group (0 hPT vs. 24 hPT; NAP, F(1, 47) = 6.84, p = 0.01; NONAP, F(1, 47) = 2.37, p = 0.12, Fig. 2A, upper panel). However, these changes in performance did not significantly differ between groups (F(1, 47) = 0.29, p = 0.58; Fig. 2B, upper panel, Changes 24 h), indicating that a post-training nap only temporarily influenced performance (i.e., during the same-day but not the next-day retest). In line with these results, the significant improvement in performance observed in the 2 groups from 8 h to 24 hPT (8 hPT vs. 24 hPT; NAP, F(1, 47) = 13.95, p < 0.001; NONAP, F(1, 47) = 28.64, p < 0.001) did not differ between NAP and NONAP conditions (F(1, 47) = 2.56, p = 0.11).

[Note that there were no significant correlations between changes in performance speed on sequence A at 8 h after interfering practice and the different sleep stages recorded during the NAP (Total sleep time, Stage 1, 2, 3, 4 and REM sleep duration, all r^2^_s_ < 0.19, p_s_ > 0.32)].

Altogether these findings suggest that a post-training nap protected performance against interference presumably triggered by the second sequence during the same day, but this effect was not significant 24 h (including a night of sleep) after initial training. Thus, the nap-dependent benefit over the day was effectively washed out following a full night of sleep, which suggests that time, including nocturnal sleep, tended to restore motor memories that were transiently challenged during the day in the absence of a post-training nap.

### Accuracy

Accuracy was very high across practice sessions as participants performed, on average, 92.8% of correct sequences. Identical to the analyses on speed above (see also Fig. 2A,B lower panels), a repeated-measures ANOVA was conducted on accuracy collected during the 4 test sessions (pT, 0 hPT, 8 hPT and 24 hPT, 4 blocks in each session) with block and session as the within-subject factors and group (NAP vs. NONAP) as the between-subject factor. Results revealed a significant session effect (F(3, 141) = 4.45, p = 0.005), indicating that accuracy changed across sessions in both groups. No significant group effect (F(1, 47) = 1.11, p = 0.29) was observed. The block by group (F(3, 141) = 0.98, p = 0.40) and block by group by session (F(9, 423) = 0.72, p = 0.69) interactions were not significant, indicating that the two groups did not differ in online changes in accuracy across the 4 blocks of practice in the various sessions. The session by group interaction was also not significant (F(3, 141) = 0.61, p = 0.61).

Inspection of the session effect (collapsed across the 2 groups) indicated that accuracy significantly decreased from pT to 0 hPT (F(1, 47) = 7.18, p = 0.01), perhaps as a side effect to the large increase in speed between these two sessions, showed stabilization at 8 h (8 hPT vs. 0 hPT, F(1, 47) = 2.03, p = 0.15) and improvement at 24 h (24 hPT vs. 0 hPT, F(1, 47) = 10.06, p = 0.002). Nonetheless, since accuracy was quite high and did not differ between groups, our interpretation of the behavioral results was based on the movement speed data presented above.

### Brain imaging results

#### Between-session changes in cerebral activity during day 1

**Figure 3:**
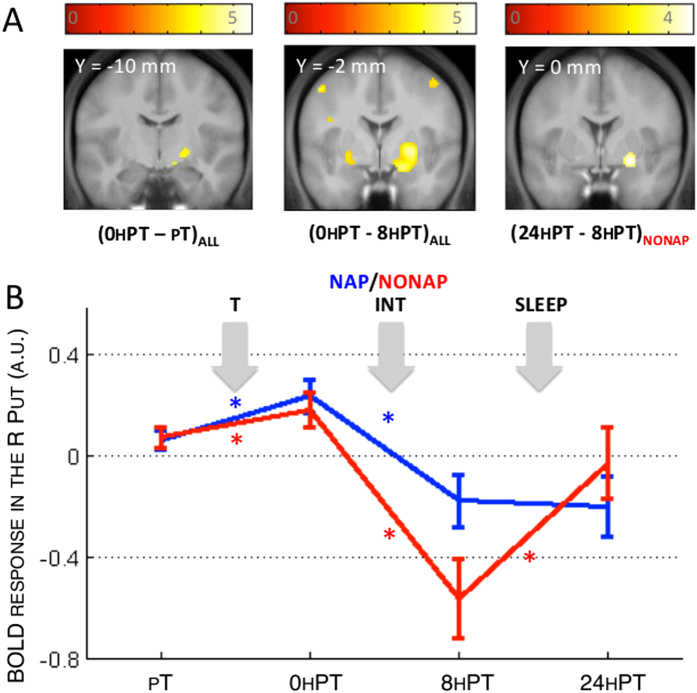
(**A**) Activity in the striatum increased as a result of training and decreased after interfering practice in both groups. Striatal activity increase was observed overnight in the NONAP group. Functional results are displayed at p_uncorrected_ < 0.001 over the mean structural image of all subjects. (**B**) BOLD response in the right putamen (PUT) across the different time points. Bars represent SEM. *Represents significant between-session changes within group (p < 0.05).

As expected, no significant main effect of group was observed on changes in cerebral activity between pT and 0 hPT, as both the NAP and NONAP participants experienced the same training procedure (Table 2.1). However, a significant decrease in activity was observed across groups, from pT to 0 hPT in the right superior parietal lobule. In contrast, activity increased from pT to 0 hPT in the right putamen and bilaterally in the sensorimotor cerebellum (lobule VI), suggesting that training on the learned sequence resulted in an increase in activity in striato-cerebello-motor areas in both groups (Fig. 3, [0 hPT - pT]_ALL_).

Changes in cerebral activity between 0 hPT and 8 hPT reflect the effects of both diurnal sleep/wakefulness and interfering practice (on sequence B) on the neural correlates related to the retention of the learned sequence (sequence A) at 8 h. Surprisingly, no significant main effect of group was observed on changes in cerebral responses between 0 hPT and 8 hPT (Table 2.2), indicating that a nap after training and before interfering practice did not significantly influence, on average, the neural correlates of memory retention at 8 h (but see *Correlation with changes in performance* section below). Interestingly, however, a large decrease of activity in motor-related cerebral areas was observed at 8 hPT as compared to 0 hPT across NAP and NONAP groups. These areas included the putamen (large bilateral activations) as well as a set of motor and parietal cortical areas. In line with our behavioral results showing significant slower performance in the NONAP as compared to the NAP group after interfering practice, data inspection revealed that striatal decrease at 8 h tended to be larger, albeit not statistically, in the NONAP group as compared to the NAP group (Fig. 3, [0 hPT–8 hPT]_ALL_).

#### Correlation with changes in performance

**Figure 4:**
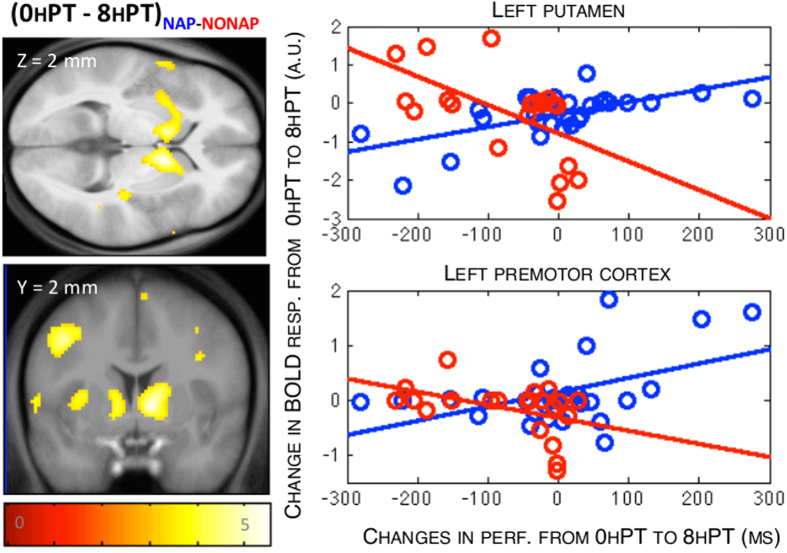
Regression between the decrease in activity in striato-cortical areas and changes in performance between 0 h and 8 hPT differed between NAP and NONAP groups. The more participants presented decrease in cerebral activity, the more their performance deteriorated from 0 hPT to 8 hPT in the NAP as compared to the NONAP group. The reverse effect was observed in the NONAP group, as illustrated on the scatter plots showing putamen and premotor cortex activity. Functional results are displayed at p_uncorrected_ < 0.001 over the mean structural image of all subjects.

We explored whether there was a relationship between the changes in cerebral activity observed between training and the 8 h retest and the corresponding changes in performance. Results revealed a main effect of group in striato-cortical areas. Specifically, the two groups differed in the relationship between activity in the striatum and a set of cortical regions, including parietal and motor areas and changes in performance from 0 h to 8 hPT (Table 3.1). Within group analyses (Table 3.2) indicated that this effect was explained by a significant relationship between the decrease in activity in a striato-cortical areas and deterioration of performance from 0 h to 8 hPT in the NAP group. In other words, the less participants presented a decrease in striato-cortical activity from 0 h to 8 hPT, the less their performance deteriorated in the NAP group. An inverse relationship was observed in striato-cortical areas in the NONAP group (Fig. 4). These results collectively suggest that although napping before interfering practice does not, on average, support significant enhancements in striato-cortical activity as compared to wakefulness (see Fig. 3, 0 hPT to 8 hPT), it modulates the link between activity in these regions and changes in performance at 8 h.

#### Between-session changes in cerebral activity between day 1 and day 2

In line with the behavioral results, no main effect of group was observed between post-training and 24 h retest (Table 4.1). However, a significant main effect of group was observed from 8 h to 24 h retest sessions in cortical areas including motor and frontal regions (Table 4.2). Within-group analyses indicated that this effect was explained by a significant overnight *increase* in activity in the putamen and the motor cortex in the NONAP group (Fig. 3, [24 hPT–8 hPT]_NONAP_), as well as significant overnight *decrease* in activity in frontal, parietal and motor areas in the NAP group. These results suggest that the memory trace undergoes distinct overnight processing, based on whether or not a nap was afforded before interfering practice. Inspection of the time course of striatal activity suggests that activity in the putamen was restored overnight in the NONAP group, as compared to the NAP group, after it was altered at 8 h (Fig. 3B).

## Discussion

The aim of the present study was to deepen our understanding of motor memory consolidation through the investigation of the neural correlates of the influence of sleep on interference to consolidation. Our results showed that a post-training nap transiently protected the memory trace against the deleterious effect of competing practice during the same day. At the cerebral level, while a post-training nap did not, *on average*, modulate brain activity, napping significantly modulated *the link* between post-interference changes in brain activity and behavior. Specifically, a smaller decrease in cortico-striatal activity after the practice of the second sequence was associated with better performance at 8 h within the NAP group. Interestingly, deterioration in performance in the NONAP group presumably mediated by interference during the first day was only transitory, as a similar level of performance was reached overnight in both groups. This overnight improvement in performance was paralleled by a restoration of the previously hindered striato-motor activity in the NONAP group. Altogether, our results offer the first evidence that the striatum, and associated cortical areas, play a critical role in (1) the overday transient sleep-facilitated reduction of susceptibility to interference and (2) the overnight restoration of previously disrupted motor memories observed over the course of consolidation.

The present behavioral results showed that performance was slower at 8 h post-training when no nap was afforded between training and interfering practice as compared to the nap group. The present results therefore confirmed that a post-training nap protects the memory trace against the negative effect of competing practice, as observed in earlier research^11^. However, interfering practice after initial motor sequence learning has been previously shown to not only disrupt memory stabilization the same day^11^, but also hinder the emergence of subsequent overnight gains in performance^11^,^23^. Contrary to these previous observations, the deterioration in performance observed over day in the present study was only transitory, as similar levels of performance were observed in both groups after a full night of sleep. Our results suggest that time, including nocturnal sleep, abolishes the transitory advantage offered by a post-training nap during the day. It remains unclear why the beneficial nap effect was only transitory and why no long-lasting effect of interference was observed at 24 h in contrast to^11^. It is possible that the difference in performance computation (i.e., mean time to perform a correct sequence vs. number of correct sequences per 30 s) or in task administration between the two studies (i.e., computerized version of the task vs. finger opposition task) may have influenced the results (for a recent discussion on the influence of different task demands and conditions in procedural memory consolidation processes, see ref. 26). In particular, it may be more difficult to significantly interfere with a highly familiar task (pressing keys on a keyboard) and automatized (consolidated knowledge of how to use a keyboard) components. Nonetheless, it is tempting to speculate that nocturnal sleep can rescue memory traces previously damaged as a result of interfering practice during the day. Interestingly, similar restorative effect of sleep on memory have recently been observed in the absence of interference^4^ but also after interference^27^ in the motor and perceptual domain, respectively. Importantly, the fact that over-day but not overnight memory consolidation processes were impaired could potentially suggest that these behavioral determinants of memory consolidation - also defined as stabilization and enhancement^1^,^28^ - are supported by distinct and independent physiological processes^28^. However, an alternative explanation that is more consistent with our brain imaging results is that these behavioral over-day and overnight phenomena share common processes that are not irreversibly altered by the competing practice taking place during the initial training day (see discussion on cerebral correlates).

Furthermore, and certainly not mutually exclusive from the interpretation offered above, the pattern of results observed in the present study between days 1 and 2 can be interpreted in the context of memory reconsolidation processes^29^. It could be speculated that the retest session taking place at 8 h post-training reactivated the motor memory that subsequently underwent reconsolidation processes^23^,^30^,^31^. In the context of this interpretation, our results suggest that the reactivation of a disrupted motor memory (in the NONAP group) triggered efficient reconsolidation processes. These findings would then indicate that efficient overnight reconsolidation could compensate for interfered over-day consolidation. Consistent with our discussion above, this would indicate that reconsolidation is modulated by the state of the memory trace that underwent consolidation processes before reactivation. We therefore argue that consolidation and reconsolidation processes share common and interacting mechanisms (but see ref. 32); however, note that the present study was not designed to test this particular hypothesis and that this interpretation does not explain the discrepancies between the present results and our previous research^11^. Future research should also characterize whether the pattern of results observed in the present study at 24 h is triggered by the 8 h (potentially reactivation) session or whether, as suggested in previous research^4^, the restorative effect of nocturnal sleep can be observed without reactivation.

Our behavioral results collectively suggest that a post-training nap transiently (i.e., the same day) protects the initially learned motor sequence against behavioral interference. Importantly, performance that was deteriorated during the day was restored overnight, suggesting that time including nocturnal sleep washes out the temporary protective effect offered by a post-training nap.

Our neuroimaging results show that, in contrast to what was observed at the behavioral level, post-training diurnal sleep did not significantly influence averaged changes in brain activity after practice of the second sequence at 8 h. Indeed, during the same-day post-interference retest, a large decrease in activity in a motor-related network, including the putamen, motor and parietal areas was observed irrespective of the napping condition. Decreases in activity in the parietal cortex is usually observed over the course of motor learning (in the absence of any interfering manipulation) and reflect the disengagement of control processes with practice^33^,^34^. In contrast, activity in the striatum and motor cortical areas has usually been described to increase or stabilize in the absence of post-training interference after periods of sleep or wakefulness^14^,^16^,^17^,^18^,^21^,^22^. Remarkably, however, the present results indicate that activity in striato-motor areas significantly decreased after interfering practice in both groups. While our study was not designed to explore the neural correlates of interference *per se* (i.e., no control group with *no interference* was included), our results suggest that the large disengagement of striato-motor activity presumably mediated by interference appears to challenge memory stabilization processes during the same day. These findings are in line with our previous work showing a critical role of the striatum in the maintenance of motor performance^6^. They are also in line with what is observed as a result of interference to motor memory *reconsolidation* during which an alteration in connectivity between the striatum and the motor cortex has been reported^35^. Interestingly, while a post-training nap did not, on average, prevent the post-interference decrease in striato-motor activity as compared to wakefulness (see Fig. 3), results of the regression analyses show that diurnal sleep significantly influenced the relationship between these changes in brain activity and behavior at 8 h. Specifically, the less participants presented a decrease in activity in striato-motor structures at 8 h, the more their performance stabilized, but only if a nap was afforded after initial training. These results suggest that diurnal sleep after initial training, as compared to wakefulness, injected inter-subject variability in over-day memory consolidation processes at both the behavioral and neural levels. However, it remains unclear why this relationship was opposite in the NONAP group and future research will be necessary to address this particular point. Altogether, the present data indicate that sleep transiently facilitates motor memory stabilization processes over the day through a modulation of cortico-striatal activity.

Importantly, the performance deterioration observed at 8 h when no nap was afforded after training was only transitory as similar levels of performance were reached in both groups after a night of sleep. Interestingly in the NONAP group, offline changes in performance at 24 h were paralleled by an overnight increase in activity in striato-motor areas. These results suggest that the previously degraded memory trace was restored overnight to post-training level and that this process was supported by a recovery of striato-motor activity. Results also indicate that the decrease in cortico-striatal activity experienced during the day after practice of sequence B is not irreversible, as activity went back to baseline levels overnight. The present results are again remarkably in line with what is observed after interference to motor memory *reconsolidation* whereby additional practice tended to restore the functional connectivity within striato-motor networks^35^. The present results speak to a particular beneficial effect of time including nocturnal sleep on this restoration process.

In summary, our results indicate that a diurnal nap prevented the decrease in activity in striatal structures presumably mediated by interference during the same day. Furthermore, time, including a night of sleep, appeared to restore both performance and striatal activity to post-training level in the NONAP group. Altogether, the present results confirm the importance of the striatal system in motor sequence memory consolidation processes^2^. Interestingly, although the hippocampus has been shown to play a critical role in motor sequence memory consolidation processes^2^, activity in this structure (and associative cortical areas) did not appear to be significantly modulated by interference. Based on the evidence that hippocampo-cortical activity is particularly linked to the emergence of *sleep-dependent enhancement* in performance^6^,^16^,^17^, we argue that preserved activity within this network triggered a nocturnal sleep-dependent boost to consolidation processes in both groups. It is however unclear whether the lack of enhancement in performance observed at 8 h in the NAP group is related to a temporary disruption of hippocampo-cortical activity during the day.

## Limitations

As mentioned in the introduction and the discussion, a limitation of the present study is the lack of control group with no interference. Accordingly, it is only assumed that the practice of the second sequence is interfering with consolidation processes of the first sequence. However, it is worth nothing that this is a reasonable assumption based on the extant literature^11^,^23^,^24^,^25^.

## Conclusions

Understanding how the brain deals with the plethora of motor tasks – which often share common attributes – necessary for everyday functioning is of the utmost importance. The results of the present study, which was the first to investigate the neural correlates of the influence of sleep on interference to motor memory consolidation, show that a post-training nap transiently protected the memory trace against the deleterious effect of interference through a modulation of activity in striatal structures during the same day. Importantly, performance that was deteriorated over the day after competing practice was rescued overnight in parallel to a restoration of cortico-striatal activity. Collectively, our results highlight how sleep modulates activity in cortico-striatal areas to facilitate the reduction of motor memory vulnerability to interference and the restoration of previously disrupted motor memories over the course of consolidation.

## Methods

### Ethics Statement

All participants gave their written informed consent to take part in the study, which was approved by the Ethics Committee of the Faculty of Medicine at the University of Liège. Procedures were performed in accordance with the approved guidelines. Subjects were compensated for their participation.

### Population

Fifty-nine young (mean age: 21.9 ± 2.2 years, 36 females), right-handed^36^, healthy volunteers were recruited by local advertisements. They had no history of medical, neurological, psychiatric or sleep-related disorders. None of the subjects were taking medications at the time of testing and all were non-smokers. None received formal training as a typist or on musical instruments requiring dexterous movements of the fingers. All the participants were moderate morning, intermediate or moderate evening chronotypes, as assessed by the morningness-eveningness questionnaire^37^. The quality of participants’ sleep in the month preceding the experimental sessions was assessed with the Pittsburgh Sleep Quality Index questionnaire^38^. Participants were instructed to respect a constant sleep schedule (according to their own rhythm ± 1 hour) during the 4 days preceding the experiment as well as over the two experimental days (i.e., 5 nights in total). Compliance to the schedule was assessed using both sleep diaries and wrist actigraphy (Cambridge Neuroscience, Cambridge, UK). The quality of participants’ sleep in the nights preceding the experimental sessions was assessed using the St Mary Hospital questionnaire^39^. All participants were also instructed to not ingest caffeine, alcohol or drugs four days before the experiment as well as during the two experimental days.

Of the 59 subjects who participated in the study, ten subjects were excluded from the analyses: two for being statistical outliers during training (performance slower than the sample average +2 standard deviations), one for not completing the retest on time because of a scanner malfunction, one for not performing any correct sequences during the last two blocks of the training session and six subjects for experiencing more than 30 s (one epoch) of sleep during the wake period (i.e., assigned to the NONAP group). The relatively high number of subjects showing more than 30 s of sleep during the NONAP episode (6 out of 29) is due to the very strict nature of the control NONAP condition (subjects were lying down in bed during 90 minutes under dim light condition and without any social interaction, see experimental procedure). Eventually forty-nine subjects were included in the final analyses: 28 subjects in the NAP group (mean age 21.5 ± 1.6 years, 19 females) and 21 in the NONAP group (mean age 22.1 ± 2.1 years, 9 females).

### Task and general experimental design

Subjects were scanned during six separate sessions [note that this protocol is part of larger design including supplemental practice sessions administered after the 6^th^ session and that are not reported in the present paper] during which they were either *trained* or *tested* on a sequential finger tapping task coded in Cogent2000 (http://www.vislab.ucl.ac.uk/cogent.php) and implemented in MATLAB (Mathworks Inc., Sherbom, MA). The task required subjects to tap on a keyboard, with their (left) non-dominant hand, a five-element finger sequence, while lying in the scanner. The sequences to perform were not displayed on the screen during testing but were explicitly taught to the participants before scanning and consisted of two types: the learned sequence (Sequence A: 4 1 3 2 4, where 1 corresponds to the index and 4 to the little finger) and the interfering sequence (Sequence B: 4 2 3 1 4). In a previous study, a control experiment showed no difference in baseline production of sequences A and B^10^. The different sessions were analogous to previous research^11^ and referred to as the pre-training test (pT, Sequence A), training (T, Sequence A), immediate post-training test taking place less than 5 minutes after training has ended (0 hPT, Sequence A) and thus before the early boost period described in the literature^4^,^12^, interfering training (INT, Sequence B), 8 h-post-training test (8 hPT, Sequence A) and 24 h-post-training test (24 hPT, Sequence A) sessions (Fig. 1). Note that as the goal of the study is to investigate the time course of consolidation processes after interference, only the data related to the ***test sessions*** are reported in the present paper (i.e., pT, 0 hPT, 8 hPT and 24 hPT, green blocks in Fig. 1).

Practice sessions were administrated with two different regimens allowing either extensive motor training (***training sessions***, 10 blocks of practice) or testing performance with a reduced amount of practice (***test sessions***, 4 blocks of practice, see Fig. 1). During ***training sessions***, i.e., training on sequence A (T) and interfering training on sequence B (INT), the task was performed in a cue-initiated manner, where the initiation of each sequence was cued by an auditory signal at a rate of 0.4 Hz (2.5 s between each cue). The training sessions consisted of 10 successive practice blocks each, audio-visually triggered by a green fixation cross, displayed on the rear-projected screen, and a tone. Each block of practice was composed of 16 cued repetitions of the sequence (sequences A and B for the training and interfering sessions, respectively) and was separated from the next practice block by 15-second rest periods that were visually indicated by a red fixation cross and a different tone.

During the ***test sessions*** (i.e., pT, 0 hPT, 8 hPT and 24 hPT), the task was performed in a self-initiated manner, i.e. participants were provided with an initial audio-visual cue (green cross accompanied by a tone) and were instructed to continuously tap sequence A, as fast and accurately as possible, until given a stop signal (red cross accompanied by a different tone). For these sessions, the task was performed in 4 successive practice blocks, each practice block being separated by 15-second rest periods. After a fixed number of key presses (i.e., 60 key presses, ideally corresponding to 12 correct sequences), the practice block automatically turned into a rest block where subjects were simply required to look at a fixation cross. Such a procedure permitted the control of the number of movements executed in each block, which is known to modulate brain activity in motor-relevant networks^40^. Accordingly, the duration of the practice blocks progressively decreased with learning as subjects became faster at performing the 60 key presses.

The particular combination of cue- and self-initiated practice was adapted from our previous work^11^. For all practice sessions, motor skill performance was assessed with **speed** (mean time to perform a correct sequence by block in ms) and **accuracy** (mean number of correct sequences by block) measures.

### Experimental procedure

One week prior to the fMRI experiment, all the subjects came to the sleep lab to perform a 90-minute habituation nap, which was monitored using standard polysomnographic recording materials and procedures (see details in next section) in the early afternoon (approximately 1 p.m.) in order to become familiar with the equipment and the sleep lab. Subjects returned to the lab one week later to perform the fMRI experiment (depicted in Fig. 1). At approximately 12 p.m. on the experimental day, each participant performed the initial session consisting of a pre-training (pT) performance test, a training (T) session and an immediate post-training (0 hPT) performance test on sequence A in the fMRI scanner. All participants were then transferred to the sleep lab where they were randomly divided into two groups according to whether they were allowed to take a 90-minute nap (NAP) or to stay in quiet wakefulness for an equivalent period of time (NONAP). The 90-minute nap and wake periods started around 1 p.m. and were monitored using polysomnography. Participants in the NONAP group were required to rest with their eyes open and to read magazines while lying on a bed under dim light conditions during the 90-minute wake period. The experimenters did not interact with the participants during the duration of the NONAP episode except if they showed signs of drowsiness (i.e., drop-out of alpha activity on occipital channels, disappearance of blinks, slow eye movements). In this case, the experimenter entered the bedroom and asked the participant if he/she was fine and encouraged him/her to go back to reading to avoid falling asleep. After this 90-minute interval, i.e., around 2:30 p.m., all subjects were scanned again while practicing the interfering sequence B (INT). At the end of the interfering training session, a psychomotor vigilance task (PVT)^41^ was administered to each subject in order to compare the level of vigilance between the sleep and wake groups. The PVT task was administered after (not before) the interfering training session in order to minimize the delay between training and interfering practice. This ensured that the practice of sequence B occurred within a two-hour post-training window (i.e., sensitivity to interference window^11^,^23^,^24^,^25^). After the PVT, the participants were then allowed to leave the lab and go back to their daily activities (with the instruction to avoid finger tapping, napping, as well as caffeine, alcohol and drug consumption) until their last visit of the day at approximately 8 p.m. for the 8 h post-training (8 hPT) scanning test session on sequence A. All the subjects spent the night at home and were asked to respect the same instructions as given for the afternoon break as well as their regular sleep schedule. The participants were then retested in the scanner around 11 a.m. the next morning on sequence A (24 hPT).

### Polysomnographic data acquisition and analyses

Habituation naps, experimental naps and wake periods were recorded with a digital sleep recorder (V-Amp, Brain Products, Gilching, Germany; bandwidth: DC to Nyquist frequency) and were digitized at a sampling rate of 500 Hz. Standard electroencephalographic (EEG) recordings were made from Fz, C3, Cz, C4, Pz, Oz, A1 and A2, with A2 used as the recording reference and A1 as a supplemental individual EEG channel. An electrode placed on the middle of the forehead was used as the recording ground. Bipolar vertical and horizontal eye movements (electrooculogram: EOG) were recorded from electrodes placed above and below the right eye and on the outer canthus of both eyes, respectively. EEG and EOG data were recorded with a 0.1 Hz low cutoff and a 30 Hz high cut-off filters. Bipolar submental electromyogram (EMG) recordings were made from the chin, filtered from 10 to 200 Hz to record muscle tone and movements. Electrical noise was filtered using a 50 Hz notch.

Polysomnographic data of the diurnal sleep (NAP) and wake (NONAP) recordings were visually scored, with 30-s epochs, by a trained sleep technician according to standard criteria^42^ using the fMRI Artefact rejection and Sleep Scoring (FASST) Toolbox^43^ (http://www.montefiore.ulg.ac.be/~phillips/FASST.html, University of Liege). To easily visualize the relevant features of sleep and wakefulness, EEG was re-referenced to an average of A1 and A2 displayed from 0.5 to 30 Hz, EOG below 10 Hz and EMG above 10 Hz using software filters.

### fMRI data acquisition and analysis

Functional MRI-series were acquired during the 6 sessions of practice of the motor task using a head-only 3T scanner (Siemens, *Allegra*, Erlangen, Germany). Multislice T2*-weighted fMRI images were obtained with a gradient echo-planar sequence using axial slice orientation (TR = 2130 ms, TE = 40 ms, FA = 90°, 32 transverse slices, 3 mm slice thickness, 30% inter-slice gap, FoV = 220 × 220 mm^2^, matrix size = 64 × 64 × 32, voxel size = 3.4 × 3.4 × 3.0 mm^3^). A structural T1-weigthed 3D MP-RAGE sequence (TR = 1960 ms, TE = 4.43 ms, TI = 1100 ms, FA = 8°, 176 slices, FoV = 230 × 173 mm^2^, matrix size = 256 × 192 × 176, voxel size = 0.9 × 0.9 × 0.9 mm^3^) was also acquired in all subjects. Head movements were minimized using a vacuum cushion.

Only imaging data from the 4 ***self-initiated test*** sessions (i.e., pT, 0 hPT, 8 hPT and 24 hPT) were analyzed [note that 2 additional self-initiated test sessions taking place after the 24 hPT session were also included in the model but the results related to these sessions are not reported in the present paper]. The 3 initial scans for each session were discarded to allow for magnetic saturation effects. Functional volumes were pre-processed and analyzed using SPM8 (http://www.fil.ion.ucl.ac.uk/spm/software/spm8/; Wellcome Department of Imaging Neuroscience, London, UK). Functional scans of each session were realigned using rigid body transformations, iteratively optimized to minimize the residual sum of squares between the first and each subsequent image separately for each session, and a mean realigned image was created. The mean functional image was coregistered to the structural T1-image using a rigid body transformation optimized to maximize the normalized mutual information between the two images. Coregistration parameters were then applied to the realigned BOLD time series. The mapping from subject to MNI space (Montreal Neurological Institute, http://www.bic.mni.mcgill.ca) was estimated from the structural image with the “unified segmentation” approach^44^. The normalization parameters were subsequently applied to the individually coregistered BOLD times series, which were then spatially smoothed using an isotropic 8-mm full-width at half-maximum (FWHM) Gaussian kernel.

The analysis of fMRI data, based on a summary statistics approach, was conducted in 2 serial steps accounting for fixed and random effects, respectively. Changes in brain responses were estimated, for each subject, by a model including the responses to the learned sequence and their linear modulation by performance speed (mean time to perform a correct sequence by block) during each testing session (pT, 0 hPT, 8 hPT and 24 hPT). The 15-second rest blocks occurring between each block of motor practice served as the baseline condition modeled implicitly in the block design. These regressors consisted of box cars convolved with the canonical hemodynamic response function. Sounds indicating the start and the end of each practice blocks, as well as errors (incorrect key press occurring within practice blocks) were also modeled, as events of no interest. Movement parameters derived from realignment of the functional volumes were also included as covariates of no interest. Movements were very limited during scanning and none of the subjects moved more than 1 voxel (average (std) translation and rotation across axis, scans, sessions and participants: 0.20 (0.24) mm and 0.17 (0.17) deg; maximum absolute movement in translation <3.4 mm and in rotation <2.7 deg). A repeated-measure ANOVA using sessions (pT, PT, 8 hPT and 24 hPT), movement type (translation and rotation) as well as axis (x, y and z) as within-subject factors and group (NAP vs. NONAP) as the between-subject factor did not show any effect of group or significant interaction between group and session, movement type or axis (all p_s_ > 0.17). High-pass filtering was implemented in the design matrix using a cut-off period of 128 seconds to remove slow drifts from the time series. Serial correlations in fMRI signal were estimated using an autoregressive (order 1) plus white noise model and a restricted maximum likelihood (ReML) algorithm.

Contrasts tested the main effect of practice of the trained sequence and its linear modulation by performance speed during each session (pT, 0 hPT, 8 hPT and 24 hPT). Supplemental linear contrasts tested the main effect of practice across all sessions and the following between-session effects: [pT vs. 0 hPT], [0 hPT vs. 8 hPT], [0 hPT vs. 24 hPT] and [8 hPT vs. 24 hPT]. The resulting contrast images were then further spatially smoothed (Gaussian kernel 6 mm FWHM) and entered in a second-level analysis, corresponding to a random effects model, accounting for inter-subject variance.

In the second level analyses, main effects of group were first explored with F-tests on the between-session contrasts described earlier. When appropriate, follow-up two-sample and one-sample t tests were then run in order to explore between- and within-group changes in cerebral responses, respectively. To assess the relationship between inter-session changes in brain activity and motor behavior, we performed a regression analysis at the second level. This analysis focused on the inter-session changes for which behavioral results differed between groups (i.e., from 0 hPT to 8 hPT). Specifically, we regressed the individual within-subject contrast images testing for the [0 hPT vs. 8 hPT] between-session difference against the corresponding changes in performance. A two-sample t tests compared these regressions between groups (NAP vs. NONAP).

The resulting set of voxel values for each contrast constituted a map of the F [SPM(F)] or the t [SPM(T)] statistic, thresholded at p < 0.001 (uncorrected for multiple comparisons). Statistical inferences were performed at a threshold of p < 0.05 after family-wise error (FWE) correction for multiple comparisons over small spherical volumes (small volume correction - SVC - procedure) located in *a priori* defined motor-related structures of interest including the striatum, hippocampus, cerebellum, motor, frontal and parietal cortices reported in published work on motor sequence learning^6^,^17^,^21^,^45^,^46^ (see list of specific coordinates in the caption of Tables 2, 3, 4). Specifically, 10 mm-radius spheres were drawn around the peak coordinates of interest reported in these papers and correction for multiple comparisons was performed, for each region of interest, across the voxels included in this sphere. Bonferroni correction was then used on the results obtained after SVC to control for the number of regions of interest per contrast. Only the results surviving both SVC and Bonferroni corrections are reported in the table (p_svc-bonf_).

## Additional Information

**How to cite this article**: Albouy, G. *et al.* Cerebral Activity Associated with Transient Sleep-Facilitated Reduction in Motor Memory Vulnerability to Interference. *Sci. Rep.* **6**, 34948; doi: 10.1038/srep34948 (2016).

## Figures and Tables

**Table 1:** Sleep and vigilance data.

	NAP	NONAP	NAP vs. NONAP
1. Questionnaires			
Pittsburgh Sleep Quality Index questionnaire			
Sleep duration	7 h 51 min ± 1 h 03 min	8 h 02 min ± 53 min	t(41) = −0.62, p = 0.53
Sleep quality^a^	4	3	t(41) = 0.22, p = 0.82^b^
St. Mary’s Hospital Sleep questionnaire			
*Night before training*			
Sleep duration	7 h 40 min ± 42 min	7 h 28 min ± 38 min	t(47) = 1.02, p = 0.30
Sleep quality^a^	4	4	t(47) = 1.53. p = 0.13
*Night before retest*			
Sleep duration	7 h 47 min ± 51 min	8 h 08 min ± 49 min	t(47) = −1.45, p = 0.15
Sleep quality^a^	4	4	t(47) = −0.09, p = 0.92
2. Actigraphic data and sleep diary (4 nights before training and 1 night before retest)			
Mean sleep duration across the 5 nights	8 h 10 min ± 59 min	8 h 28 min ± 58 min	Night by group: F(4, 188) = 0.18, p = 0.9
Sleep duration - night before training	7 h 38 min ± 57 min	7 h 52 min ± 51 min	F(1, 47) = 0.81, p = 0.37
Sleep duration - night before retest	8 h 21 min ± 1 h	8 h 40 min ± 55 min	F(1, 47) = 1.21, p = 0.27
3. Psychomotor Vigilance Task (PVT)			
Mean reaction time	311.89 ± 39.01 ms	293.68 ± 35.67 ms	t(46) = 1.66, p = 0.10^c^
4. Daytime sleep characteristics			
Total Recording Time	1 h 31 min ± 1 min	—	—
Total Sleep Time	1 h 00 min ± 18 min	—	—
Sleep efficiency^d^	66.73% ± 19.87%	—	—
Stage 1 Sleep Latency	10 min ± 4 min	—	—
Time awake	21 min ± 15 min	—	—
Time in Stage 1 Sleep	8 min ± 4 min	—	—
Time in Stage 2 Sleep	38 min ± 12 min	—	—
Time in Stage 3 Sleep^e^	4 min ± 3 min	—	—
Time in Stage 4 Sleep^e^	10 min ± 13 min	—	—
Time in REM Sleep^e^	6 min ± 7 min	—	—

^a^Median score.

^b^Note that PSQI data of 6 subjects (3 NAP and 3 NONAP subjects) were never returned by the participants.

^c^Note that PVT data of one subject of the NAP group were not recorded.

^d^Sleep efficiency was computed as the percent of time asleep relative to the total time in bed (specifically, from lights off to lights on).

^e^5/28 subjects did not present Stage 3 sleep; 10/28 did not present Stage 4 sleep and 10/28 did not reach REM sleep.

**Table 2:** Between-session changes in cerebral activity within the first day.

Area	x mm	y mm	z mm	Z	p_svc-bonf_
**1. pT vs. 0 hPT**					
***Main effect of group***(***F-Test***)					
No significant responses					
[***pT***** > *****0 hPT***]_***ALL***_					
Right Superior Parietal Lobule	34	−44	66	3.51	**0.015**
[***0 hPT***** > *****pT***]_***ALL***_					
Left Cerebellar Lobule VI	−8	−68	−20	4.02	**0.001**
Right Cerebellar Lobule VI	10	−68	−16	3.61	**0.005**
Right Globus Pallidus/Putamen	20	−10	−4	3.60	**0.006**
**2. 0 hPT vs. 8 hPT**					
***Main effect of group***(***F-Test***)					
No significant responses					
[***0 hPT***** > *****8 hPT***]_***ALL***_					
Right Putamen	18	2	−16	4.79	**0.000**
	26	0	−6	4.14	**0.001**
	22	4	−4	3.98	**0.005**
	26	−4	−4	4.51	**0.000**
Left Putamen	−22	16	−8	3.71	**0.005**
Right Premotor Cortex	44	2	56	4.19	**0.001**
Right Superior Parietal Lobule	26	−62	48	4.39	**0.000**
Left Superior Parietal Lobule	−20	−74	48	3.91	**0.002**
[***8 hPT***** > *****0 hPT***]_***ALL***_					
No significant responses					

Brain responses reported are significant after correction for multiple comparisons over small volume of interest (svc) and Bonferroni correction for the number of regions of interest per contrast (bonf). List of coordinates used for small volume correction: striatum 22 −6 −2 mm, 22 4 −14 mm^17^; cerebellum ±12 −76 −18 mm^6^; premotor cortex 38 6 62 mm^45^; superior parietal cortex 28 −44 68 mm^46^, ±22 −64 48 mm^17^.

**Table 3:** Regression between changes in cerebral activity and performance from 0 h to 8 hPT.

Area	x mm	y mm	z mm	Z	p_svc-bonf_
**1. Between-group differences in regression**					
***Main effect of group***(***F-Test***)					
*Significant responses in striato-cortical networks (see detailed T-tests*)					
[***0 hPT***** > *****8 hPT***] ***×***[***NONAP***** > *****NAP***]					
No significant responses					
[***0 hPT***** > *****8 hPT***] ***×***[***NAP***** > *****NONAP***]					
Left Putamen	−10	10	−2	4.78	**0.000**
	−24	12	6	4.22	**0.001**
	−14	6	6	3.85	**0.003**
	−26	−20	−4	3.71	**0.005**
Right Putamen	10	4	2	4.72	**0.000**
Left Premotor Cortex	−42	2	40	4.15	**0.001**
Right Superior Parietal Cortex	30	−44	66	3.94	**0.002**
**2. Within-group regression effects [0 hPT > 8 hPT]**					
***−NAP***(***regression with performance deterioration***)					
Left Putamen	−6	4	−6	4.45	**0.000**
Right Putamen	4	2	−2	4.05	**0.001**
Right Superior Lateral Frontal Gyrus	28	36	46	3.60	**0.007**
**+*****NAP***(***regression with performance improvement***)					
No significant responses					
***−NONAP***(***regression with in performance deterioration***)					
No significant responses					
**+*****NONAP***(***regression with performance improvement***)					
Left Putamen	−10	10	−2	3.70	**0.005**
Right Putamen	12	8	4	3.68	**0.005**
SMA	−6	−6	50	3.63	**0.006**

Brain responses reported are significant after correction for multiple comparisons over small volume of interest (svc) and Bonferroni correction for the number of regions of interest per contrast (bonf). List of coordinates used for small volume correction: striatum ±10 12 −4 mm, −30 −14 −10 mm^17^; premotor cortex ±34 4 42 mm^21^; supplementary motor area ±2 0 54^46^; superior parietal cortex 28 −44 68 mm^46^; frontal cortex 22 38 46 mm^46^.

**Table 4:** Between-session changes in cerebral activity between days.

Area	x mm	y mm	z mm	Z	p_svc-bonf_
**1. 0 hPT vs. 24 hPT**					
***Main effect of group***(***F-Test***)					
No significant responses					
**2. 8 hPT vs. 24 hPT**					
***Main effect of group***(***F-Test***)					
*Significant responses in striato-cortical networks (see detailed T-tests*)					
[***8 hPT***** > *****24 hPT***] ***×***[***NONAP***** > *****NAP***]					
No significant responses					
[***24 hPT***** > *****8 hPT***] ***×***[***NONAP***** > *****NAP***]					
Pre-Supplementary Motor Area	2	20	50	4.02	**0.002**
Right Premotor Cortex	42	6	54	4.08	**0.001**
Left Middle Frontal Gyrus	−48	30	16	3.66	**0.006**
***Within-group effects***					
***NAP***					
[***8 hPT***** > *****24 hPT***]					
Left Middle Frontal Gyrus	−48	28	20	4.02	**0.002**
Left Superior Parietal Lobule	−26	−58	52	3.74	**0.005**
M1 (Medial)	10	−38	66	3.66	**0.006**
Left IPS	−44	−48	46	3.64	**0.007**
[***24 hPT***** > *****8 hPT***]					
No significant responses					
***NONAP***					
[***8 hPT***** > *****24 hPT***]					
No significant responses					
[***24 hPT***** > *****8 hPT***]					
Right Putamen	24	0	−10	4.00	**0.002**
Left M1/S1	−22	−32	62	3.77	**0.004**

Brain responses reported are significant after correction for multiple comparisons over small volume of interest (svc) and Bonferroni correction for the number of regions of interest per contrast (bonf). List of coordinates used for small volume correction: striatum 24 0 −12 mm^17^; premotor cortex 38 6 62 mm^45^; pre-supplementary motor area ±6 18 58 mm^46^; medial M1 ±6 −34 60 mm^46^; M1/S1 −16 −32 54^46^; superior parietal cortex 22 −64 48 mm^17^; intraparietal sulcus ±50 −54 42 mm^46^; frontal cortex ±48 24 14 mm^6^.
